# Are Polyunsaturated Fatty Acids Implicated in Histaminergic Dysregulation in Bipolar Disorder?: AN HYPOTHESIS

**DOI:** 10.3389/fphys.2018.00693

**Published:** 2018-06-12

**Authors:** María E. Riveros, Mauricio A. Retamal

**Affiliations:** ^1^Centro de Fisiología Celular e Integrativa, Facultad de Medicina, Clínica Alemana Universidad del Desarrollo, Santiago, Chile; ^2^Center of Applied Ecology and Sustainability, Santiago, Chile; ^3^Department of Cell Physiology and Molecular Biophysics, Center for Membrane Protein Research, Texas Tech University Health Sciences Center, Lubbock, TX, United States

**Keywords:** BAS, bipolar disorder, fatty acids, histaminergic system, omega-3, PUFAs

## Abstract

Bipolar disorder (BD) is an extremely disabling psychiatric disease, characterized by alternate states of mania (or hypomania) and depression with euthymic states in between. Currently, patients receive pharmacological treatment with mood stabilizers, antipsychotics, and antidepressants. Unfortunately, not all patients respond well to this type of treatment. Bipolar patients are also more prone to heart and metabolic diseases as well as a higher risk of suicide compared to the healthy population. For a correct brain function is indispensable a right protein and lipids (e.g., fatty acids) balance. In particular, the amount of fatty acids in the brain corresponds to a 50–70% of the dry weight. It has been reported that in specific brain regions of BD patients there is a reduction in the content of unsaturated n-3 fatty acids. Accordingly, a diet rich in n-3 fatty acids has beneficial effects in BD patients, while their absence or high levels of saturated fatty acids in the diet are correlated to the risk of developing the disease. On the other hand, the histamine system is likely to be involved in the pathophysiology of several psychiatric diseases such as BD. Histamine is a neuromodulator involved in arousal, motivation, and energy balance; drugs acting on the histamine receptor H3 have shown potential as antidepressants and antipsychotics. The histaminergic system as other neurotransmission systems can be altered by fatty acid membrane composition. The purpose of this review is to explore how polyunsaturated fatty acids content alterations are related to the histaminergic system modulation and their impact in BD pathophysiology.

## Introduction

Bipolar disorder (BD) is a prevalent psychiatric disease characterized by recurrent episodes of depression and elevated mood (mania), intermingled with periods of normal mood (euthymia) (Phillips and Kupfer, 2013). Pharmacological treatments consist of lithium for prophylaxis, as well as anticonvulsants and antipsychotics for acute treatment in manic episodes, in conjunction with psychotherapy (Geddes and Miklowitz, 2013). All these pharmacological approaches have side effects that negatively impact on the life quality of patients. Is well-recognized that BD constitutes a significant burden for the patient (reduced life expectancy and increased risk of suicide), because of that considerable amount of efforts has been conducted in this field in order to improve the patient’s quality of life (Vogelzang et al., 2012). One major problem to find new and better treatments for BD symptoms is the lack of a good animal model that recapitulates the core feature of this disorder: spontaneous oscillations between manic, euthymic, and depressive-like behavioral phenotypes. Currently, most of the research is based on animal models that show manic-like behavior without depression. Nevertheless, BD patients spend more time in the depressed phase than in their manic phases (Judd et al., 2002). Depression in BD is usually refractory to treatments and has to be treated differently than monopolar depression, which uses drugs like tricyclic antidepressants because these can trigger the switch from depression to mania (Gijsman et al., 2004). It would be crucial to differentiate what distinguishes bipolar depressed from a monopolar depressed state and which are the alterations that lead to switching from depressed to manic state and destabilize the euthymic state in BD patients. BD has a strong genetic component being transmitted within families for several generations (Craddock and Sklar, 2013). Genetic alterations for BD have contributed to understanding the characteristic features of the disease, and have helped to determine that BD shares a wide range of features with psychiatric diseases such as autism, monopolar depression, and schizophrenia. The genetic bases of this disease seem to be multifactorial instead of caused by a single gene mutation. However, in spite of this genetic component, this disease can be drastically modulated by the fat diet composition, been unsaturated fatty acids (e.g., omega-3) diet composition crucial. Additionally, dysfunctions in the neuromodulator systems are also common in various psychiatric disorders. Thus, extensive research about the relationship between serotonin, noradrenaline, and dopamine on different mental diseases has been done, but the role of histamine in psychiatric illnesses has been much less studied (Baronio et al., 2014). Moreover, the link between the histaminergic system and fatty acids appears to have a lot of therapeutic potential against BD. In this review, we will discuss the recent evidence regarding the link between fatty acid intake, histaminergic system modulation, and the BD improvements.

## Fatty Acids and Bipolar Disorder

The amount of fatty acids in the brain corresponds to a 50–70% of its dry weight (O’Brien and Sampson, 1965b), and the composition of fatty acids is very important for its right function. In particular, polyunsaturated fatty acids (PUFAs) correspond to 20% of brain weight (O’Brien and Sampson, 1965a), and among them, docosahexaenoic acid (DHA, C22:6n-3) and arachidonic acid (AA, C20:4n-6) are the most abundant (O’Brien and Sampson, 1965a). Both, AA and DHA (and their metabolites), participate in many important brain functions such as acting as intracellular second messengers, neurotransmission, gene transcription, among other brain processes (Hibbeln et al., 1989). Additionally, fatty acids at the cell membrane can directly interact with membrane proteins, determining their structure and function. Thus, they can determine the membranes fluidity, lateral pressure, bilayer thickness, and surface charge distribution (Stillwell and Wassall, 2003). There is a high specificity in lipid–protein interaction, for example, slight changes in fatty acid conformation can alter their interaction with proteins, as for example the loss of one double bond when a molecular simulation is conducted replacing DHAn-3 with DPAn-3, generate changes in the balance of attraction and repulsion forces that determinate the bending force in a monolayer which is translated in the lateral pressure profile in a bilayer which can alter protein functional conformational changes as for example G protein-coupled receptors activation (Gawrisch and Soubias, 2008). Therefore, the presence of DHA in the membrane can modulate neurotransmission systems signaling, contributing in this way to brain function. Moreover, PUFAs can affect brain functioning by activating many kinds of receptors and cell signaling pathways and also by modulating the endocannabinoid system. These potential mechanisms of action of n-3 fatty acids in brain physiology have been revised in detail by Bazinet and Layé (2014).

Accordingly, to the exposed above, epidemiological studies have revealed that fatty acids composition of the diet is associated with mental health (Messamore et al., 2017). As for example, populations with a lower incidence of BD have higher rates of seafood consumption (Noguchi et al., 2013). While a study comparing intake of PUFAs in bipolar patients versus a non-psychiatric control population showed that bipolar diagnosed individuals have a reduced intake of PUFAs [eicosapentaenoic acid (EPA) (n-3), docosahexaenoic acid (DHA) (n-3), arachidonic acid (AA) (n-6), and docosapentaenoic acid (DPA)] and higher intake of saturated fats (Evans et al., 2014). Moreover, not just the saturated versus PUFAs consumption is relevant, but also very important is the ratio among n-3 and n-6 PUFAs consumption, for more details about this topic (see Messamore et al., 2017). Nevertheless, diet composition does not constitute an estimate of the fatty acid composition in the brain, in order to establish a functional relation of fatty acid composition and brain physiology alterations an estimation of brain PUFAs content is needed. A non-invasive way to estimate brain fatty acid composition is the erythrocyte fatty acid composition, because erythrocyte DHA and cortical DHA content are correlated (Carver et al., 2001). The amount of DHA measured in the plasma membranes of erythrocytes in BD patients is lower compared to the DHA in control patients (Chiu et al., 2003; McNamara and Welge, 2016). These results are in agreement with animal studies showing that diet deficient in n-3 fatty acids alter monoamine systems in limbic structures known to control mood (Chalon, 2006; Jiang et al., 2012), which provides a possible link between fatty acid intake and psychiatric disorders as BD.

Moreover, fatty acids in the brain could be linked to neuroinflammatory processes, known to be at the base of many psychiatric diseases and BD. AA is an n-6 PUFA which initiates a signaling cascade by conversion of a part of the released AA to bioactive eicosanoids. Cyclooxygenase 2 (COX2) transforms AA into the prostaglandin E2 (PGE2) initiating neuroinflammation processes (Rapoport, 2008). Post-mortem brains of BD patients have increased levels of AA synthesizing enzymes (Kim et al., 2011). It has been proposed an AA hypothesis to explain the effect of mood stabilizers used to treat BD. Mood stabilizers affect AA cascade but not in a single point of action, for example, lithium and carbamazepine decrease AA levels by reducing PLA2 expression, and therefore AA levels. Additionally, downstream signaling of AA is reduced by lithium, carbamazepine, valproate, and lamotrigine because they inhibit COX2 expression. AA signaling cascade as a target for BD symptomatology treatment could also help to explain the positive action of an n-3 fatty acid enriched diet in BD, because n-3 fatty acids oppositely regulate AA and DHA bioavailability (Rao et al., 2007) they avoid overreactions in AA cascade (Lands, 2015). Accordingly, antidepressants with high risk to produce mania as fluoxetine and imipramine increase brain AA concentration and cPLA2 activity (Rao et al., 2006; Lee et al., 2010), but bupropion or lamotrigine which do not induce AA production, are not associated to the risk of inducing mania.

It has been recently reported using molecular simulation that the presence of DHA in the membrane accelerates the rate of oligomerization of dopamine D2 receptor and adenosine A2A receptor, being higher when the DHA content in the membrane is “healthy like” compared to a “disease like” reduced content of DHA in the membrane (Guixà-González et al., 2016). This effect is mediated by increased lateral mobility of receptors and favorable interaction between DHA and proteins as well as favorable interactions among DHA tails and the rest of the membrane lipids, which induces segregation of DHA-coated proteins in enriched DHA domains. Therefore, the increased diffusion and concentration of proteins in these domains increases the chances of protein–protein interactions and accelerates oligomerization (Guixà-González et al., 2016). Nevertheless, information regarding the effect other PUFA as for example n-6 PUFA on protein–protein interaction is lacking, in order to link this molecular effect of DHA on dopamine and adenosine oligomerization to behavioral effects related to n-3/n-6 ratio it would be necessary to evaluate the effect of membrane enrichment with an n-6 PUFA.

In animal models, the use of controlled oil supplementation in the diet has been extremely helpful to elucidate how lipid composition impacts BD related symptomatology. For example, supplementation of the diet for two generations with either fish oil (rich in n-3 fatty acids) or a diet rich in trans fatty acids (TFA) from pregnancy to adulthood, induced different brain lipid composition in the second generation (Trevizol et al., 2015). The second generation of rats fed a diet rich in TFA had a reduced n-3 fatty acids composition in the brain, together with an increased hyperactivity response to amphetamine injection compared to fish oil group. The brains of fish oil supplemented animals have also higher levels of BDNF and reduced reactive oxygen species (ROS) compared to animals fed with the TFA rich diet, showing that a high level of trans-fatty acids in the diet may facilitate the development of neuropsychiatric conditions, including BD. Moreover, a diet high in TFA resulted in a reduced expression of dopamine transporter in the hippocampus and increased rates of amphetamine self-administration, in the second generation of rats (Trevizol et al., 2015). Fatty acids composition of the brain can also be related to addictive behavior which is notably higher in BD population (Regier et al., 1990). These data suggest that symptomatology of BD, as well as altered pathways involved in it, could be modulated by diet supplementation with PUFAs, and the data emphasize the important role of PUFAs in BD etiology.

Similarly, to the example exposed above, chronic amphetamine administration in mice, induces an increased locomotor response to amphetamine after withdrawal, this is known as sensitization. Sensitized mice have manic and depressive behaviors together with circuitry alterations related to BD (Pathak et al., 2015). In accordance, an increase in the AA:DHA ratio induced by the dietary deficiency in DHA, increases the behavioral sensitization to amphetamine (McNamara et al., 2008). AA:DHA ratio positively correlates with amphetamine-induced locomotor activity, DHA deficiency also induces an increase in DHA extracellular concentration in the brain that is positively correlated with AA:DHA ratio and amphetamine-induced locomotor activity (McNamara et al., 2008).

Furthermore, trials using n-3 PUFA diet enrichment have shown variable but promising results for the treatment of BD. For example, supplementation with n-3 PUFA as adjunctive therapy has a positive effect in bipolar depression, significantly bigger than placebo, but the same methanalysis concludes that this is not true for manic symptoms (Sarris et al., 2012). However, some reports show less confident results, as for example the metanalysis performed by Montgomery et al showing that the design and execution of the trials must be improved in order to reach conclusive results, for example, the duration of the trial has to be of minimum 3 months to achieve changes in brain fatty acids composition, the conclusion of this metanalysis is that of the analyzed works just one have positive results but just in depressive symptoms, again, with no effect of n-3 supplementation on manic symptoms (Montgomery and Richardson, 2008).

Moreover, caution should be taken when interpreting results of n-3 diet enrichment in patients and designing trials. One of the issues to take in account is the impact of the disease in the patient’s style of life. BD diagnosed population has higher rates of unemployment and divorce, familial conflicts, and poor diet habits (Morselli et al., 2004). Considering this, just to adhere to a trial could have a positive effect, because of the increase in the structure in their lifestyle. It should also be considered, that omega-3 supplementation can have a positive effect in overall health, as it has been reported to be beneficial for metabolic alterations and cardiovascular health (Carpentier et al., 2006; Sudheendran et al., 2010). Therefore, it should be taken in to account that together with their effect on brain functioning omega-3 supplementation could improve bipolar symptoms trough by increasing well-being and improving cellular and systemic functioning in general.

A better understanding of the consequences of n-3/n-6 ratio in BD symptomatology could help to improve treatments targeting lipid composition and its molecular consequences.

## Histaminergic Neurotransmission System

Neuronal histamine is synthesized by neurons in the tuberomammillary nucleus (TMN) of the posterior hypothalamus (Brown et al., 2001). In mammalian brain, histamine acts (mainly) as an excitatory neurotransmitter and has been implicated in various central functions, as for example, arousal modulation, motivation, energy balance, motor behavior, and cognition (Benarroch, 2010; Torrealba et al., 2012). The histaminergic system has been implicated in energy balance in many ways (Tabarean, 2016). For example, the regulation of food consumption, energy expenditure (Yoshimatsu, 2006) and thermoregulation (Tabarean et al., 2012). Histamine effects are mediated by the activation of four G protein-coupled receptors: H1R, H2R, H3R, and H4R (Panula and Nuutinen, 2013). H1R and H2R activation have excitatory effects in post-synaptic neurons (Haas et al., 2008). H3R is the most abundant in the CNS, and it can be either an autoreceptor, located on the dendrites and axonal varicosities of histaminergic neurons, where it modulates the release of histamine or it can be a heteroreceptor able to regulate the release of other neurotransmitters. In addition, H4R is expressed mainly on immune cells, and it modulates the immune response during the inflammatory state (Deesch et al., 2005). In the CNS the function and location of H4Rs are not completely clear (Schneider and Seifert, 2016). Histamine acting through H1 and H2 receptors increases arousal, most likely by increasing cortical and thalamic activity, as well as increasing the activity of other arousal nuclei as the orexinergic lateral hypothalamic nucleus, the noradrenergic locus coeruleus, or the cholinergic nuclei in the basal forebrain and the pons (Khateb et al., 1990). Histaminergic neurons activity is strongly correlated to active waking and their activity is drastically reduced during slow wave sleep (Takahashi et al., 2006). During awakening histaminergic signaling increase levels of arousal necessary to increase performance during goal-directed behavior, increasing vigor and persistence, as part of a motivated state. During the active phase, these histaminergic neurons promote a higher activity and excitability in cortex and thalamus, regulating, in turn, motor activity, facilitating, accelerating, and improving motor responses. For example, motor balance and coordination are modulated by histaminergic inputs to the cerebellum, acting via H2 receptors, directly in the cerebellar nuclei, the output of the cerebellum (Zhang et al., 2016). Also, histamine seems to modulate central vestibular-mediated motor reflexes and behaviors through H2 receptors in the lateral vestibular nucleus neurons which control muscle tone and vestibular reflexes, injection of histamine in this site improves motor behavior (Li et al., 2016). Furthermore, the basal ganglia, a functional network implicated in motor control, receives histaminergic projections, and histamine release during the wake period (Cumming et al., 1991), a component of the basal ganglia particularly well-innervated by histaminergic fibers is the striatum, which expresses a high density of histamine receptors (Hill and Young, 1980) supporting the idea of an important histaminergic modulation of striatal, and therefore motor, function. Motor abnormalities are part of the neurological soft signs present in BD euthymic patients, and motor hyperactivity is characteristic of mania as motor retardation characterizes depression. Thus, motor function is affected in all phases of the disease and histaminergic alterations could underlie these abnormalities.

It is interesting to note that mast cells, that promote inflammation and allergic response by releasing histamine, are also present in the brain and mast cells degranulation and non-neuronal histamine release normally promote wakefulness and motivated behavior as suggested by the increased delta power and reduced food seeking motivated behavior in mast cell-deficient mice compared to wild-types, importantly this mice have also more anxious and depressive behaviors than wild-type mice (Chikahisa et al., 2013). It is known that n-3 PUFAs have an antiallergenic effect (Willemsen, 2016) and as mentioned before, can improve BD symptoms, it is possible that both effects are related to the fact that mast cell activation is inhibited by n-3 PUFAs (Wang et al., 2015). Similarly, vitamin D that has also been suggested to improve BD symptoms (Sikoglu et al., 2015) participates in mast cell stabilization (Liu et al., 2017).

Histamine H3R are a key factor in histaminergic functions because they exert an autoinhibitory effect over histaminergic cells, controlling their activity and histaminergic release and synthesis. Thus, histaminergic neurons firing can be inhibited by H3 agonists injected into the TMN (Flik et al., 2011) The H3R mediated histaminergic autoinhibitory effect depends on calcium release from intracellular stores; in fact, preventing intracellular calcium storage uploading with thapsigargin reduce H3R mediated autoinhibition of firing frequency (De Luca et al., 2016). Also, the autoinhibitory effect of histamine through H3R is impaired in depolarized cells (De Luca et al., 2016). In addition, depolarization of histaminergic neurons increases intracellular calcium through voltage-gated calcium channels (Uteshev and Knot, 2005). H3R has been suggested as a target for drugs to treat some aspects of neurodegenerative and psychiatric diseases as cognitive dysfunctions, sleepiness, or overweight (Sander et al., 2008). Interestingly, H3 antagonists are promising as potential antipsychotic drugs (Mahmood, 2016). Accordingly, clobenpropit an H3 antagonist injected systemically or into the hippocampus reverse the immobility and cognitive impairment in a rat depression model, and this effect depends on histamine release and action over H1 and H2 (Femenía et al., 2015). Thus, the histaminergic system is promising as a target for treating depressive and manic states in BD.

Additionally, histamine through H3 can inhibit melanin-concentrating hormone (MCH) (Parks et al., 2014). MCH has been implicated in depression because injections of MCH in the dorsal raphe induce a depressive phenotype in rats (Lagos et al., 2011), that could be due to the inhibition of serotoninergic neurons by MCH (Torterolo et al., 2015). Accordingly, MCH receptor 1 antagonists have antidepressant actions (Borowsky et al., 2002). On the other hand, MCH is also related to sleep, and its levels are higher during sleep and reduced in active wakefulness, as shown in rodents (Pelluru et al., 2013) and in humans (Blouin et al., 2013). Moreover, MCH microinjections in different areas can induce sleep and reduce latency to REM, and increase the length of REM periods, in rats and cats (Torterolo et al., 2009; Monti et al., 2015). These characteristics are similar to characteristics of sleep in depression (Palagini et al., 2013). Thus, through its action in the MCH system by H3 activation, high levels of histamine can reduce sleep, and low levels of histamine could be related to an increased MCH activity and depressive behavior. Therefore, reduction in histamine levels could induce a depressed state, by increasing MCH together with the putative impairment in histamine-derived functions such as motor activity, motivated arousal, cognitive functions, and others.

Another aspect of histamine that merits attention regarding a possible role for a histaminergic alteration in BD is the role of histamine in addiction and in particular its role in alcoholism. There is a very important comorbidity between BD and alcoholism: 50% of BD patients are alcoholics (Frye and Salloum, 2006). Rats bred for alcohol preference, have a denser histaminergic innervation through the brain, and higher histamine levels than non-alcohol-preferring rats (Lintunen et al., 2001). Also, alcohol-preferring rats have a reduced expression of H3 receptors in their motor cortex, nucleus accumbens, and CA1 area of the hippocampus (Lintunen et al., 2001). In a comparable way, post-mortem brains of BD patients have a reduced H3 expression in the hippocampus (Jin et al., 2009). Response of H3 expression to stress, sleep deprivation, inflammation, and oxidative stress is worthy of exploration, as well as the response of alcoholic animals to amphetamine in order to evaluate whether the genetic predisposition to alcohol ingestion and related alterations in the histaminergic system could be associated with a genetic predisposition to develop BD or similar disorder.

As mentioned, the histaminergic neurotransmission system is implicated in arousal and behavioral activation and is necessary for a normal unfolding of motivated behaviors. Bipolar patients have an altered response to motivational stimuli. It has been hypothesized that the extreme fluctuations in behavioral activation that patients have between mania and depression is related to a dysregulated and hypersensitive behavioral approach system (BAS) (Urošević et al., 2008). BAS is a system related to behavioral activation for the approach to reward; the prefrontal cortex has an essential role in this system, and it is functionally altered in BD. One of the PFC reported to be altered in bipolar patients is the subgenual region, which has an increased metabolic activity during mania and a decreased metabolic activity during depression (Drevets et al., 1997). Interestingly, this region corresponds to the infralimbic area of the prefrontal cortex (ILC), which is the main excitatory input to the TMN. ILC activation induces histamine release, and the increase of arousal during motivated behaviors, such as food searching behavior, depends on the increase in histamine release directed by the ILC (Riveros et al., 2015). Thus, the behavioral alterations observed in BD as excessive engagement in motivated behaviors and arousal during mania, as well as the decreased motivation and arousal during depression, could be explained by alterations in histamine transmission as well as by the functional changes in regions involved in the disease.

## Proposed Model for Fatty Acid-Induced Protection of Alterations in Histaminergic Transmission System Underlying Bipolar Disorder

As mentioned before, the histaminergic system has been implicated in energy balance, food consumption, energy expenditure, and thermoregulation. Mitochondria and Na^+^/K^+^ ATPase activity are the main controllers of energy availability in the cells. The interplay between the histaminergic system and these players is therefore very likely to influence the energy expenditure in BD patients.

**FIGURE 1:**
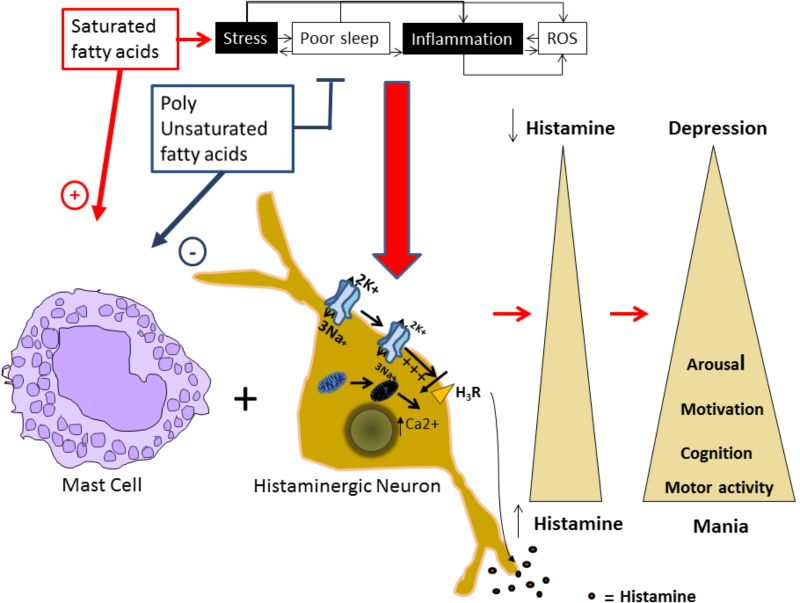
Proposed model for alterations in the histaminergic transmission system underlying bipolar disorder. Factors as stress, inflammation, increased levels of reactive oxygen species (ROS), and sleep alterations are known to induce and or worsen BD episodes, and are risk factors for developing the disorder. In our proposed model, these factors can induce a molecular environment that can increase intracellular Ca^2+^ levels and depolarize the plasmatic membrane in histaminergic neurons. In turn, these processes could affect the H3 histaminergic auto receptor affecting the autoregulation of Histaminergic neurons, reducing Na^+^/K^+^ ATPase activity and inducing mitochondrial damage. This damage leads to intracellular Ca^+^ increase and membrane depolarization that inhibits the H3 receptor, leading to an increased HA release. Additionally, saturated fatty acids promote mast cells degranulation and consequently histamine release, while polyunsaturated fatty acids (PUFAs) inhibit it. Released histamine by both means, acting on it targets will induce increased motor activity, arousal, and motivated behaviors, characteristic from a manic phenotype. On the contrary, all these behaviors will be reduced in depression in parallel with reduced histamine levels.

Alterations in the histaminergic system can lead to BD development and/or its worsening and an increase in PUFAs intake could reduce the vulnerability to develop BD. Here, we proposed that the protective effect of PUFAs could be mediated by the modulation of mitochondrial function and Na^+^/K^+^ ATPase activity in the histaminergic system (**Figure 1**).

### The Na^+^/K^+^ ATPase Role in Mood Disorders

Na^+^/K^+^ ATPase is a protein that actively transports three molecules of Na^+^ and two molecules of K^+^ against its concentration gradient and, therefore, is particularly determinant in setting the level of neural activity. Its activity can be increased by inflammation in peripheral neurons (Wang et al., 2015), and it has been suggested that NA^+^/K^+^ ATPase dysfunctions may be involved in mood disorders (Christo and el-Mallakh, 1993; Traub and Lichtstein, 2000). For instance, the Na^+^/K^+^ ATPase activity in schizophrenic patients is diminished in the erythrocyte membrane of both unipolar and bipolar depressed patients, compared to healthy controls (Rybakowski and Lehmann, 1994). These results showed no differences between bipolar and unipolar depressed patients suggesting that alterations in Na^+^/K^+^ ATPase are not an endophenotype for BD, but more likely a general marker of mental health risk (Rybakowski and Lehmann, 1994). However, BD patients erythrocytes have a lower level of activity of NA^+^/K^+^ ATPase in manic and depressed states compared to euthymic BD patients (Looney and el-Mallakh, 1997).

Furthermore, post-mortem brains of bipolar individuals have reduced expression of Na^+^/K^+^ ATPase α2 in the temporal cortex (Rose et al., 1998). Accordingly, seem to be genetic associations between BD and variants encoding Na^+^/K^+^ ATPase α1, α2, and α3 subunits (Goldstein et al., 2009). Additionally, Myshkin (Atp1a3Myk/+; Myk/+) mice that carry a mutation in the Atp1a3 gene, results in a 36–42% reduction in total Na^+^/K^+^ ATPase activity in the brain (Clapcote et al., 2009), shown behavioral alterations comparable to mania [hyperactivity, risk-taking behaviors, and reductions in rapid eye movement (REM) and non-REM sleep] (Kirshenbaum et al., 2011). Furthermore, these alterations respond to lithium and valproic acid treatment (Kirshenbaum et al., 2011), suggesting that reduced Na^+^/K^+^ ATPase activity could be a common mechanism in behavioral abnormalities and bipolar subjects that respond to lithium and valporic acid.

The over activated state observed in BD can also be induced pharmacologically by blocking the Na^+^/K^+^ ATPase. Ouabain is a digitalis-like compound (DLC) that bounds to Na^+^/K^+^ ATPase inhibiting the transport of Na^+^ and K^+^ across the plasma membrane (Nesher et al., 2007). Altering Na^+^/K^+^ ATPase activity in the brain with an intracerebroventricular (ICV) injection of ouabain induces hyperactivity in rats (Brüning et al., 2012). This hyperactivity is reduced by administration of mood-stabilizing drugs or antipsychotics (El-Mallakh et al., 2006). Additionally, ouabain induces the release of synaptosomes from the cerebral cortex (Bersier et al., 2005). The (*n*-methyl-D-aspartate) NMDA receptor inhibitor MK-801 inhibits the ouabain-induced neurotransmitter release, suggesting that ouabain action could be mediated by the activation of NMDA receptors (Cousin et al., 1995). Furthermore, the expression of NMDA receptor subunits NR2A, NR2B, and NR2D in the cerebral cortex and hippocampus is increased by inhibition of Na^+^/K^+^ ATPase (Bersier et al., 2008), and an up-regulation of NMDA-evoked current in rat hippocampus neurons has been shown (Zhang et al., 2012). Ouabain inhibits glutamate uptake, because its transport depends on the Na^+^ and K^+^ gradients generated by Na^+^/K^+^ ATPase, and glutamate transporter physically interacts with Na^+^/K^+^ ATPase to regulate glutamatergic transmission (Rose et al., 2009) and in consequence, ouabain enhances glutamatergic neurotransmission (Nguyen et al., 2010). NMDA receptor activity regulates signal pathways related to protein translation, including ERK1/2, Akt, and mTOR, which play critical roles in synaptic plasticity regulated by the glutamatergic system (Sutton and Chandler, 2002; Gong et al., 2006). Ouabain-induced activation of mTOR signal pathway and protein synthesis could be mediated by the activation of NMDA receptor system. Ouabain injected in the brain also induces oxidative stress (Riegel et al., 2010), which is related to mood disorders, inhibits citrate synthase activity (Freitas et al., 2010), and reduces the expression of brain derived neurotropic factor (BDNF) (Jornada et al., 2010). Furthermore, ouabain activates tyrosine hydroxylase (Yu et al., 2011). All these responses resemble some aspects of the pathophysiology of mania. Therefore, injection of ouabain in brains of rodents has been established as an accepted protocol to induce behavioral and physiological alterations used as a model of mania (el-Mallakh et al., 1995).

### Effects of PUFAs on Na^+^/K^+^ ATPase Activity and Its Relationship With Mood Disorders

The unsaturated/saturated fatty acid ratio in the brain, has been observed to modulate Na^+^/K^+^ ATPase activity, being higher in animals fed with oils rich in unsaturated fats compared with animals fed with a diet reduced in unsaturated fats (Srinivasarao et al., 1997). Thus, for example, the Na^+^/K^+^ ATPase activity is altered by diabetes and can be partially restored after a fish oil diet (rich in n-3 fatty acids) (Gerbi et al., 1998). Accordingly, to these results, it has been shown that TFAs in the diet increases membrane rigidity and reduces the activity of Na^+^/K^+^ ATPase in the striatum of rats (Dias et al., 2015; Trevizol et al., 2015). Additionally, it has been observed that affinity of the α1 isoform for ouabain positively correlates with the total amount of n-6 fatty acids (Gerbi et al., 1999). These results suggest an important interaction between membrane fatty acids composition and Na^+^/K^+^ ATPase activity. Furthermore, the n3/n6 ratio has consequences in cognitive functions, as for example, rats fed with safflower oil which is high linoleate (n-6) have cognitive impairments when compared with rats fed the perilla oil which is high in alphalinoleate (n-3) (Yamamoto et al., 1987). This may be related to altered lipid composition of membranes and reduced Na^+^/K^+^ ATPase activity, in safflower oil fed rats (Tsutsumi et al., 1995). In the same line of evidence, rats fed with linseed oil show higher levels of DHA in the synaptic membranes of the brain and therefore an increased n-3/n-6 fatty acid ratio. This ratio with a higher proportion of n-3 fatty acids increases membrane fluidity, Na^+^/K^+^ ATPase activity, and serotonin levels in the brain (Sugasini and Lokesh, 2015). Fluidity is not the only physical property of the membrane affected by lipid composition that can have an effect on Na^+^/K^+^ ATPase activity. For example, the hydrophobic thickness of the membrane is also an important parameter in the pump function (Cornelius, 2001). It is determined by the carbon number in the acyl chain but also depends on the saturation of the fatty acids and their interaction with cholesterol (Cornelius, 2001), so the saturated/unsaturated ratio can also disturb the matching of the hydrophobic portion of the protein with the hydrophobic thickness of the membrane affecting by this way the pump activity (Cornelius, 2008).

**FIGURE 2:**
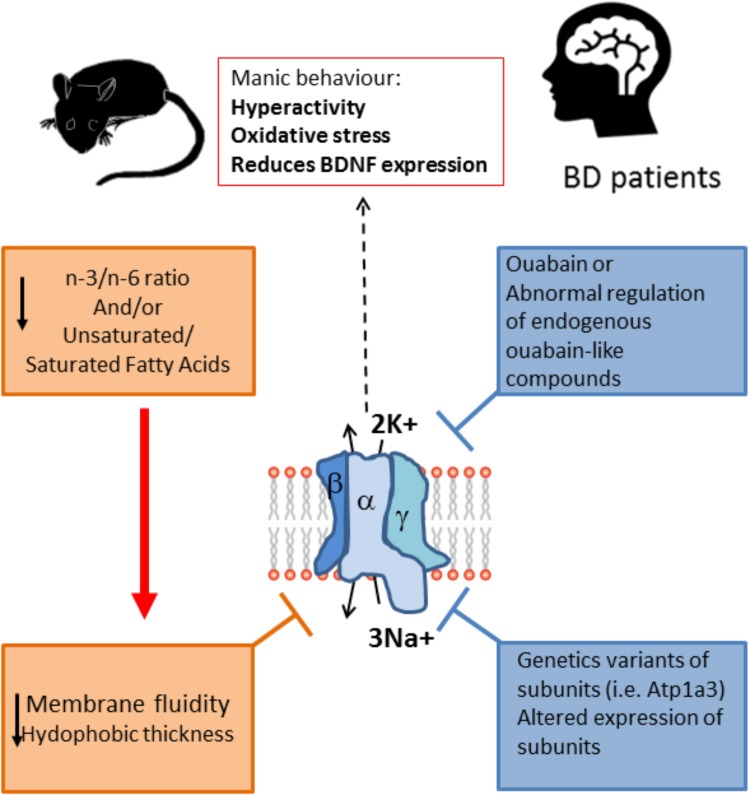
Na^+^/K^+^ ATPase activity is lower in BD patients and animal models of mania. The enzyme activity is dependent on the fluidity of the membrane, which is lower when the unsaturated/saturated fatty acids ratio at the membrane is decreased, as is the case of BD patients. Moreover, the alteration in the n3/n6 ratio also can modify the membrane fluidity and in turn decrease the Na^+^/K^+^ ATPase activity. This alteration in lipid composition could be related to the reported lower activity of the enzyme in patients, and it is likely that the lowered activity of the enzyme is related to the symptomatology of BD because reducing the activity of the enzyme pharmacologically with ouabain or by the genetic alteration of the enzyme (Atp1a3 mutant) induces a manic phenotype, with behavioral changes related to mania together with increased oxidative stress and reduction in BDNF expression. Furthermore, some BD patients have been reported to express variants of the enzyme, and also expression of the enzyme is altered in key regions of the limbic system. Altogether the evidence points toward a role for Na^+^/K^+^ ATPase in the etiology of BD. Unsaturated/saturated fatty acids ratio at the membrane also affects de hydrophobic thickness of the membrane which in turns can affect the pump activity.

It is important to note that the inclusion of PUFAs in the diet can affect neurotransmission by modulating neurotransmitter reuptake and improves the cholinergic transmission in the brain, that has an important role in cognition (Willis et al., 2009). The fatty acids composition of the diet can influence behavior and furthermore have an impact in BD symptomatology and development (**Figure 2**). Contrary to the effects of consuming PUFAs, a diet high in saturated fats is a risk factor for various mental health problems including depression and cognitive dysfunction (Sánchez-Villegas et al., 2011). In rats fed a high-saturated fat diet, a reduced H1R binding density in many brain areas was observed (Wu et al., 2013). Interestingly, the reduction of H1R binding densities in some of these areas (substantia nigra and caudate putamen) was prevented by supplementing the HF diet with n-3 polyunsaturated DHA, and also prevented the negative effect of HF in cognitive function. H1R expression is reduced in depressed patients, while omega-3, specifically DHA, levels in serum and red blood cells membranes is reduced in bipolar and major depression patients, with a greater deficit in BD patients (McNamara et al., 2010). In consequence, the evidence suggests that coincident alterations in histaminergic system and lipid composition in depression could be causally linked. Furthermore, histamine clearance (which is essential to avoid excessive histamine activity) is a process dependent on astrocyte reuptake of histamine, which in turn dependents on Na^+^/K^+^ ATPase activity and is sensitive to ouabain, thus it could have manic like behavioral consequences (Perdan-Pirkmajer et al., 2010; Yoshikawa et al., 2013) (**Figure 2**).

## Mitochondria and BD

**FIGURE 3:**
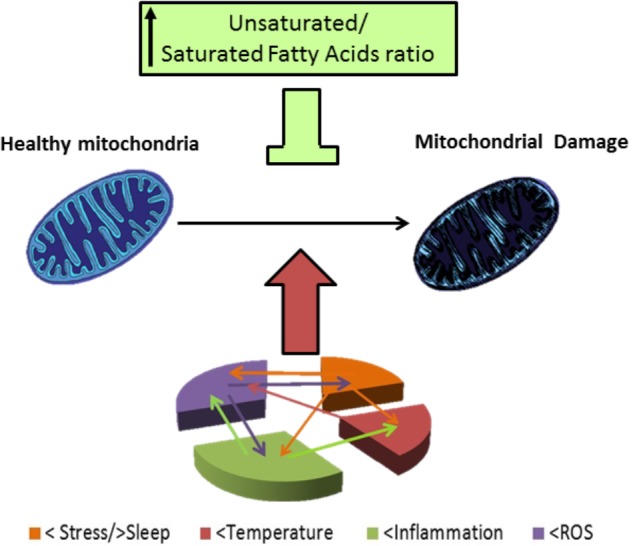
Mitocondrial damage in BD patients. BD patients have altered response to stress and frequently they have a story of life stress events. Also, there are alterations in sleep, mainly reduced sleep at night in BD patients. Stress and poor sleep can induce mitochondrial damage and induce temperature alterations as well as increase inflammation and oxidative stress (level of ROS), temperature has been reported to be increased in BD patients, and high temperature can induce mitochondrial damage. BD patients also have shown to be in a chronic inflammatory state; which; can induce mitochondrial damage, an increased temperature and oxidative stress. ROS are elevated in BD patients and this elevated ROS can worsen the inflammatory state and stress outcome, oxidative stress, and also damage mitochondria. Damaged mitochondria in BD patients have an altered morphology, reduced respiration rates, polymorphisms and deletions in DNA. In consequence of their altered function BD patients have reduced high energy phosphates levels and reduced pH, and their altered mitochondrial function, can in turn increase oxidative stress, neuro-inflammation, and have an impact in stress response. Lipid composition of mithochondrial membrane affect the organelle response to stress, in particular, high levels of PUFAs are protective against different types of stress involved in pathophysiology of BD.

Mitochondrial function is critical to neuronal physiology and survival. In spite of its traditional role as the cellular energy generator, mitochondria have other roles (Duchen, 2004). For example, they can decode intracellular signals, in particular, calcium ions (Ca^2+^) increases (Clapham, 2007). In 2000, Kato presented the mitochondrial dysregulation hypothesis for BD which was suggested by a reduction in phosphocreatine in the frontal lobe of patients with BD observed with magnetic resonance spectroscopy (Kato et al., 1992, 1994). It was shown that BD patients have a lower pH (7.01) in the brain compared to healthy controls (7.05) (Kato et al., 1998). Also, BD patients have higher levels of lactate in cerebrospinal fluid compared to a matching sample of healthy individuals (Regenold et al., 2009) as well as in the brain of bipolar patients, where lactate levels where find to be higher in the anterior cingulate cortex and caudate of BD patients by using 2D proton magnetic resonance spectroscopic imaging (Chu et al., 2013). Lactate is a product of extra-mitochondrial glucose metabolite, usually elevated in resting (no exercising) individuals with mitochondrial dysfunction (Regenold et al., 2009). Furthermore, BD among subjects with mitochondrial diseases is almost 20 times higher (16–21% prevalence) compared to the general population. For example, chronic progressive ophthalmoplegia (CPEO) which is an hereditary mitochondrial disease, sometimes has comorbidity with BD and depression (Suomalainen et al., 1992). Also, Plog 1 (mitochondrial DNA polymerase) is one of the causative genes for CPEO; the transgenic mice (mPolg1 Tg) with a forebrain-specific expression of a mutant Plog 1 gene has characteristics that make it a putative model for BD (Kasahara et al., 2006). Additionally, mitochondria isolated from brains of these transgenic mice have enhanced Ca^2+^ uptake rate (Kubota et al., 2006). Since not all BD patients have Plog 1 mutations, Kubota et al; evaluated candidate genes that were altered both in mPlog1 Tg mice and BD human patients and found that mitochondrial peptidyl-prolyl *cis–trans* isomerase (CypD) gene, was consistently downregulated in mPolg1 Tg mices and BD patients. CypD is a component of the mitochondrial permeability transition pore, using N1M811 an inhibitor of CypD ameliorates the mPolg1 Tg mice behavioral phenotype. CypD sensitizes the brain mitochondria to the transition pore, and its inhibition by CsA or CypD absence improves the complex I-related mitochondrial function and increases mitochondria stability against Ca^2+^ stress (Gainutdinov et al., 2015). In fact, mitochondria of mPolg1 Tg mices have an enhanced Ca^2+^ sequestration rate (Kubota et al., 2006). In their recent review Morris et al., evidenced that mitochondrial function in BD is higher during mania and decreased in depression, and propose that this phasic dysregulation of mitochondrial function is central to BD pathophysiology (Morris et al., 2017). Also, stress and sleep deprivation have been pointed to have a role in BD. These issues could be in part linked to their damaging effect at the level of mitochondrial function (**Figure 3**). Additionally, risks factors associated with BD such as stress and sleep restriction have been reported to induce alterations in mitochondrial function. For example, prenatal restraint stress causes a decrease in BDNF mRNA levels, an increase in ROS, and malfunctions in mitochondrial metabolism among other consequences, that can be prevented feeding mothers with a DHA supplemented diet (Feng et al., 2012). Additionally, chronic sleep restriction results in mitochondrial dysfunction in the prefrontal cortex of mice, indicated by morphological alterations and reduction in ATP level (Zhao et al., 2016). Also, chronic sleep deprivation increases oxidative stress, as it increases lipid peroxidation and formation of protein carbonyls as well as oxidation of DNA (Olayaki et al., 2015). Plus, total sleep deprivation or short-term partial sleep deprivation have been shown to induce inflammation (elevated C reactive protein) in healthy adult subjects (Meier-Ewert et al., 2004). However, mitochondrial dysfunction is not only related to BD; it is present in early pathological states in neurodegenerative diseases, as for example in Alzheimer’s, Parkinson’s, and Huntington’s disease, as well as in ischemic stroke, and also in aging brain (Al Shahrani et al., 2017).

### Role of PUFAs on Mitochondria Function

Docosahexaenoic acid improved mitochondrial function in animal models of aging and neurodegenerative diseases (Eckert et al., 2013). Animals supplemented with fish oil, which is rich in long-chain n-3 PUFAs, show an increased PUFA content in their mitochondrial membranes. Following these changes in membrane composition, increase in mitochondrial respiration rate and/or Complex IV (cytochrome c oxidase) activity have been observed (McMillin et al., 1992). PUFA supplementation in the diet has also been reported to increase lipid oxidation and reduce energy efficiency in mitochondria, and to reduce oxidative stress, together with an increase in mitochondriogenesis in skeletal muscle of PUFA supplemented rats (Cavaliere et al., 2016). Feeding mices with an n-3 PUFA enriched diet, decrease oxidative stress in cariomiocites, dependent on mitochondrial adaptations, with an increased activity of GR. Therefore, it is clear that PUFAs content in mitochondrial membrane protects the organelle in confronting stress, possibly improving the BD patient’s outcome and moreover reducing the risk of developing the disease. Histamine can induce calcium oscillations in mitochondria and increase mitochondrial permeability trough H2 receptor activation (Luo et al., 2013). Chronic stress induces mitochondrial damage (Sonei et al., 2017) and leads to the activation and opening of the mitochondrial transition pore (Kuchukashvili et al., 2012). The neuronal histaminergic system is known to be involved in acute psychological stress (Lkhagvasuren and Oka, 2017) and chronic psychological stress (Ito et al., 1999), histamine levels are increased by stress, therefore, they could be promoting effects of stress in mitochondrial permeability. Plus, histamine released by mast cells during stress is also involved in stress effects in impaired glucose tolerance and reduced sleep acting on H1 receptors (Chikahisa et al., 2017). Accordingly, while PUFAs are a protection factor in the development of BD, Histaminergic system dysregulation, that can be induced by stress and saturated fatty acids in the diet, could participate in generating the damage observed in BD and could underlie behavioral alterations associated to BD symptomatology.

## Conclusion

Stress, poor sleep, inflammation, and oxidative stress are factors that have long been implicated in neuropsychiatric diseases; they can interact and potentiate each other promoting an environment that alters neuronal physiology. Some of the alterations are related to the neuronal energy machinery focused in the mitochondria and Na^+^/K^+^ATPase, with consequences in the intracellular Ca^2+^ level and membrane potential. These could particularly be relevant in BD. These molecular and cellular alterations could modify evermore the neuronal circuits that are altered in BD. We propose that altered histaminergic transmission could result from the altered environment produced by stress, poor sleep, inflammation, and oxidative stress. Fatty acid in the diet and consequent fatty acid composition of biological membranes can reduce the damaging effects of this pathoetiological block at both cellular and neurotransmission level. The effects of this neuropsychiatric damage inducing block on the histaminergic transmission system and/or its interaction with fatty acid supplementation are worth exploring in animal models of mania and depression. The H3 autorregulatory system of histaminergic transmission appears to be strongly related to behavioral changes induced by stress, reduced sleep, inflammation, and oxidative stress. Based on our review, we propose that manic state is characterized by high levels of histaminergic transmission while depression is characterized by low levels of histaminergic transmission.

## Author Contributions

MER wrote the manuscript and did the figures. MAR edited the manuscript and figures.

## Conflict of Interest Statement

The authors declare that the research was conducted in the absence of any commercial or financial relationships that could be construed as a potential conflict of interest. The handling Editor is currently co-organizing a Research Topic with one of the authors MAR, and confirms the absence of any other collaboration.
